# Experimental modeling of Alzheimer's disease: Translational lessons from cross‐taxon analyses

**DOI:** 10.1002/alz.70273

**Published:** 2025-05-26

**Authors:** Konstantin B. Yenkoyan, Maria M. Kotova, Kirill V. Apukhtin, David S. Galstyan, Tamara G. Amstislavskaya, Tatyana Strekalova, Murilo S. de Abreu, Vergine A. Chavushyan, Lee Wei Lim, Longen Yang, Denis B. Rosemberg, Allan V. Kalueff

**Affiliations:** ^1^ Neuroscience Laboratory, COBRAIN Center Yerevan State Medical University after M. Heratsi Yerevan Armenia; ^2^ Neuroscience Program Sirius University of Science and Technology Sochi Russia; ^3^ Institute of Translational Biomedicine St. Petersburg State University St. Petersburg Russia; ^4^ Institute of Experimental Medicine, Almazov National Medical Research Centre Ministry of Healthcare of Russian Federation St. Petersburg Russia; ^5^ Scientific Research Institute of Neurosciences and Medicine Novosibirsk Russia; ^6^ Research Institute of General Pathology and Pathophysiology Moscow Russia; ^7^ Graduate Program in Health Sciences Federal University of Health Sciences of Porto Alegre Porto Alegre Brazil; ^8^ Department of Biosciences and Bioinformatics, School of Science Xi'an Jiaotong‐Liverpool University Suzhou China; ^9^ Suzhou Municipal Key Laboratory of Neurobiology and Cell Signaling, School of Science Xi'an Jiaotong‐Liverpool University Suzhou China; ^10^ Laboratory of Experimental Neuropsychobiology, Department of Biochemistry and Molecular Biology, Natural and Exact Sciences Center Federal University of Santa Maria Santa Maria Brazil; ^11^ Graduate Program in Biological Sciences: Toxicological Biochemistry Federal University of Santa Maria Santa Maria Brazil; ^12^ The International Zebrafish Neuroscience Research Consortium (ZNRC) Slidell Louisiana USA

**Keywords:** Alzheimer's disease, animal models, cross‐taxon analyses, evolutionary psychiatry, translation medicine

## Abstract

Alzheimer's disease (AD) is a severely debilitating neurodegenerative disease with a rapidly increasing global prevalence, poorly understood causes, and no efficient treatments. Experimental models are valuable for studying AD pathogenesis, including amyloid beta and tau accumulation, synaptic dysfunction, and neuroinflammation. While no model fully reproduces the disease, we take an evolutionary biology approach to discuss available models across taxa, from mammals (rodents, primates) to zebrafish, *Drosophila melanogaster*, and *Caenorhabditis elegans*. Evaluating their strengths and limitations provides insight into disease mechanisms and may refine research strategies for improved diagnostics and therapeutic screening. Traditional models have significantly contributed to AD research, yet their translational limitations highlight the need for physiologically relevant alternatives. Integrating humanized rodent models, zebrafish, organoids, and induced pluripotent stem cell–based systems—along with advances in bioengineering and genetic editing—may offer a more comprehensive framework to bridge the gap between preclinical research and clinical application.

**Highlights:**

- Experimental models across rodents, primates, zebrafish, fruit flies, and worms provide key insights into Alzheimer's disease (AD).
- Cross‐taxon comparisons assess strengths and weaknesses in AD models.
- Evolutionary biology approaches refine experimental strategies for AD research.
- Diverse animal models improve understanding of AD pathogenesis.
- Cross‐species models enhance diagnostics and therapeutic strategy development.

## Graphical abstract

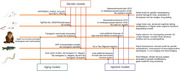

## 1 INTRODUCTION

Dementia represents one of the most common brain deficits, and affects > 55 million people globally.^1^ It is also a hallmark symptom of various severely debilitating neurodegenerative disorders, such as Alzheimer's disease (AD).^2^ Markedly impairing the quality of life, AD is a highly prevalent brain disorder whose global incidence is expected to triple by 2050,^3^ with survival estimated as < 5 years after a dementia diagnosis^4^ and 6 to 6.5 years after AD diagnosis.^5^, ^6^ AD is characterized by progressive neurodegeneration and the formation of neurofibrillary aggregates and amyloid beta (Aβ) plaques,^7^, ^8^ accompanied by synaptic dysfunctions and overt behavioral and cognitive deficits.^9^, ^10^

**FIGURE 1 alz70273-fig-0001:**
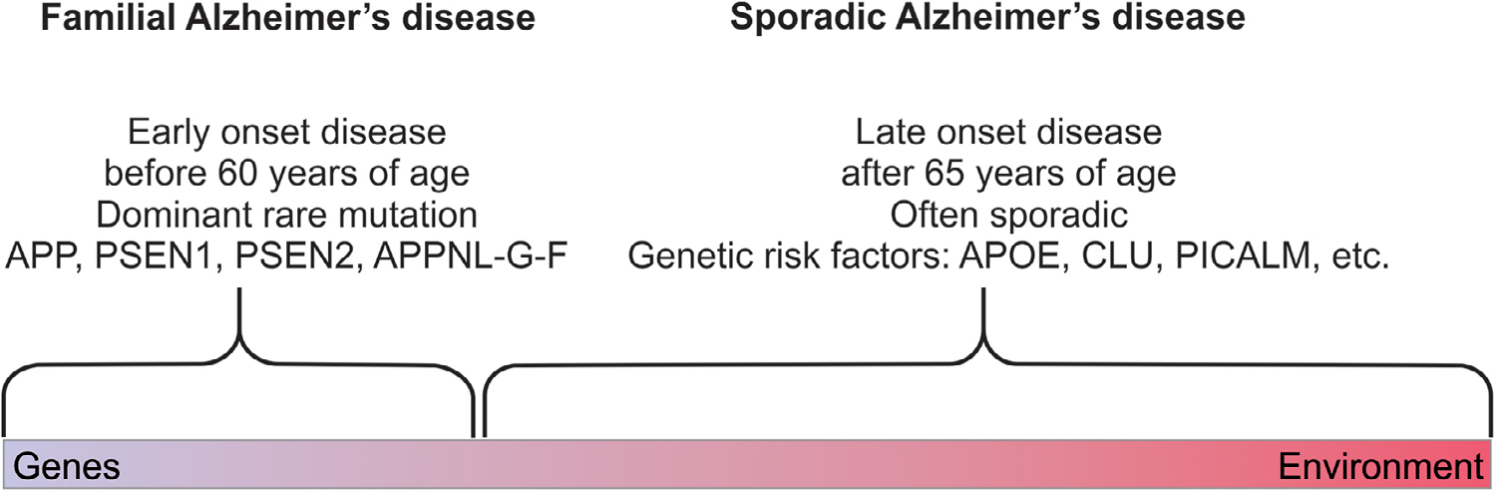
The multifactorial nature of Alzheimer's disease (AD). Early familial AD, most often caused by mutations in *APP*, *PSEN1*, or *PSEN2* genes, accounts for 1% to 5% of AD cases. The exact causes of late‐onset sporadic AD (sAD), which accounts for > 95% of AD cases, remain largely unknown, but may involve complex gene x environment x aging interactions. Both forms of AD lead to the formation of amyloid plaques (Aβ) and neurofibrillary abnormalities, oxidative stress, synaptic dysfunction, neuroinflammation, neurodegeneration, and ultimately cognitive decline and motor and other behavioral deficits.

There are presently neither preventative strategies nor disease‐modifying treatments available for AD, and its etiology remains poorly understood,^6^, ^7^, ^8^ with molecular alterations likely occurring decades prior to the onset of cognitive decline.^11^ While the early‐onset familial (autosomal dominant) AD accounts for < 5% of all cases and is associated with mutations in the *AMYLOID PRECURSOR PROTEIN* (*APP*), *PRESENELIN* *1* (*PSEN1*), and *PRESENELIN* *2* (*PSEN2*) genes,^12^ the majority (> 95%) of AD cases have late onset with no apparent familial linkage, presenting as sporadic AD (sAD, Figure 1)^13^, ^14^ whose etiology remains unclear.

The sAD subtype involves multiple disparate aging‐related pathogenic mechanisms, such as metabolic disorders, neurotrauma, neurotoxicity, hypertension, vascular damage, smoking, psychological stress, and vitamin deficiencies.^15^ There is also some genetic component in sAD, because the presence of one or two alleles of the *APOLIPOPROTEIN* *E* (*APOE*) gene increases the disease risk up to 10‐fold.^13^, ^14^, ^16^ Mounting evidence suggests that sAD results from a non‐linear pathophysiological cascade, initiating in early life and manifesting as neurodegeneration and dementia later in life.^7^, ^8^ Indeed, its key biomarkers, such as Aβ in the cerebrospinal fluid (CSF), deviate from normality ≈ 25 years before symptom onset, whereas tau protein levels and hippocampal volume changes emerge at ≈ 15 years, and cognitive impairments becoming apparent ≈ 5 years, before the onset of clinical symptoms.^12^

While genetic rodent models have significantly advanced our understanding of AD, they predominantly represent familial AD and may not fully recapitulate the complex, multifactorial nature of sAD. However, the emerging “AD+” models integrate aging‐related mechanisms, metabolic disturbances, and comorbidities, offering a more physiologically relevant representation of human pathogenesis. Taxonomic analysis may provide deeper insights into the robustness of biomarkers in identifying this heterogeneity, thereby advancing the translational potential of both familial and sAD models for clinical applications. These aspects are explored further below, as we discuss the strengths and limitations of various AD models based on rodents, primates, zebrafish, and invertebrates (a visual summary of our findings is presented in the Graphical Abstract).

## 2 MODELS OF AD

### 2.1 Rodent models of AD

While mouse and rat models each offer unique advantages for studying AD, they also complement one another in providing a more comprehensive view of the disease pathology. Mice, with their genetic manipulability, are invaluable for understanding the molecular mechanisms of AD, whereas rats, with their more complex behaviors and larger brain size, are better suited for detailed behavioral and neuroanatomical studies. Thus, together, these models provide a robust platform for investigating the diverse pathologies of AD, including amyloid and tau deposition.

**FIGURE 2 alz70273-fig-0002:**
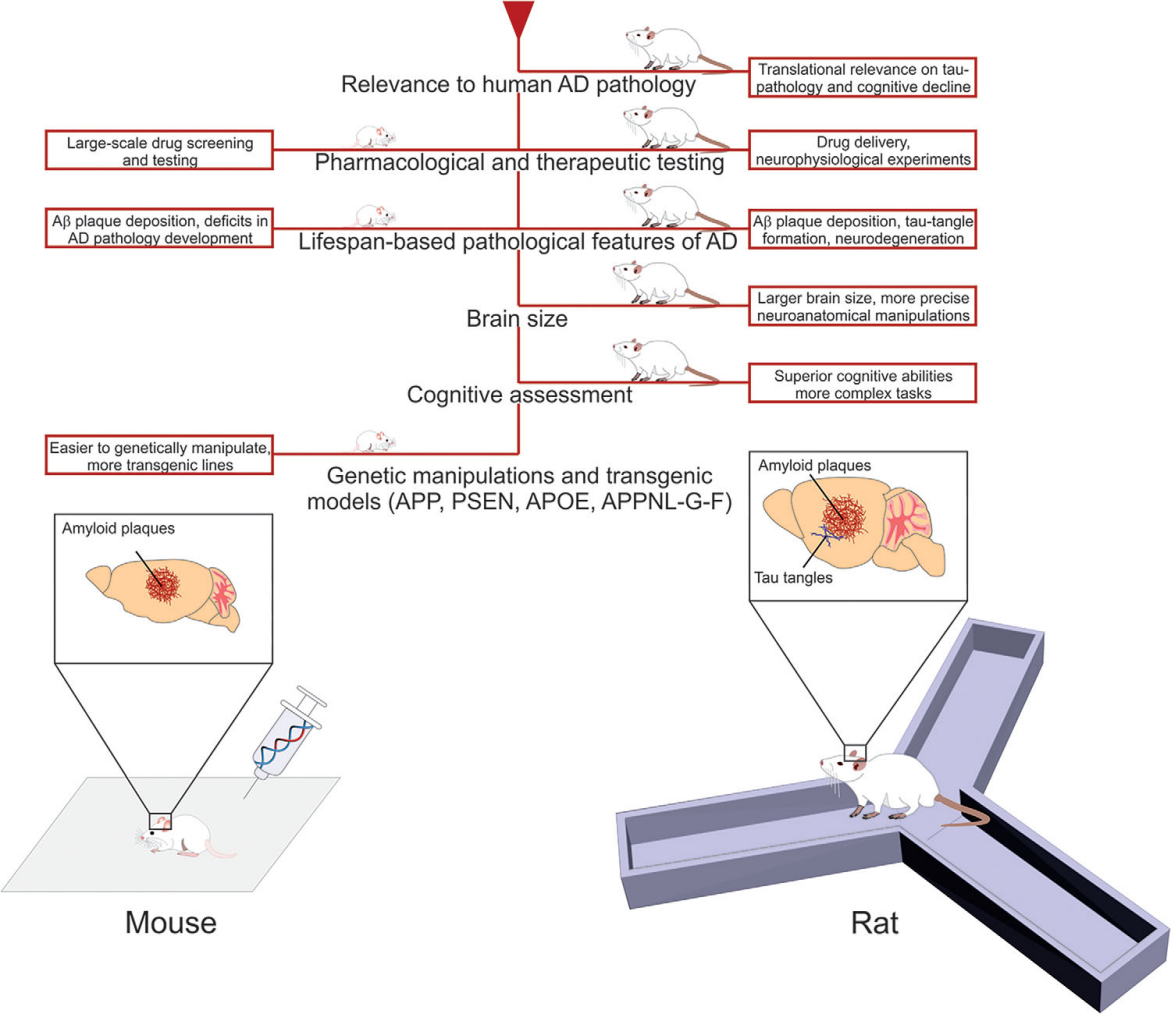
Comparison of selected animal model species presently used in Alzheimer's disease (AD) research (top panel). Bottom panel compares features of mouse and rat models of AD, emphasizing their relevance. Both models demonstrate amyloid beta (Aβ) plaque deposition, a core pathological feature of AD. Mouse models enable focusing on drug screening and genetic/transgenic models, whereas rat models with larger brain size enable more precise neuroanatomical manipulations and advanced cognitive assessments, with a greater capacity for neurophysiological experiments.

Animal models, especially rodent based, are widely used to study AD and its underlying mechanisms.^2^ Although mice are presently most widely used in this field due to their genetic manipulability, physiological homology with humans, and well‐established tools for behavioral testing (Figure 2, Table 1), rat models have recently gained increasing popularity. Their even closer evolutionary affinity with humans, more sophisticated behavioral repertoire, and larger brain size facilitate more accurate neuroanatomical investigations of AD pathology.^17^ These features allow rats to provide a more nuanced assessment of symptoms — such as learning and memory impairments — that are central to AD.^18^, ^19^ Thus, together, both rodent models provide a robust platform for investigating the diverse pathologies of AD, including Aβ and tau deposition.

**TABLE 1 alz70273-tbl-0001:** Selected vertebrate models of AD.

Model	Evoked abnormalities	References
***Genetic***		
PDAPP mice	Aβ accumulation, memory impairment	^20^
Tg2576mice	Aβ accumulation, memory impairment	^21^
TgAPP 23 mice	Aβ accumulation, memory impairment	^22^
Ob/ob mice	Anxious behavior, absence of long‐term potentiation in the hippocampus, and increased tau phosphorylation	^23^
5xFAD mice	Aβ accumulation, memory impairment, motor dysfunction, age‐related axonopathy, and neuroinflammation	^24^, ^25^, ^26^
3xTg‐AD mice	Aβ accumulation, memory impairment, motor dysfunction, age‐related axonopathy, neuroinflammation, and accumulation of phosphorylated tau	^27^
EFAD mice	Aβ accumulation, neuroinflammation, tau pathology, and neuronal plasticity disruption	^28^
McGill‐R‐Thy1APP rats	hyperactivation of hippocampal neurons	^29^
TgF344‐AD rats	Aβ accumulation, memory impairment, apoptosis, accumulation of phosphorylated tau, aberrant hippocampal microvessels	^30^, ^31^
OXYS rats	Aβ accumulation	^32^
***Aging related***		
Old mice	Aβ accumulation, impaired learning, increased apoptosis in the brain,	^33^, ^34^
***Pharmacological***		
AlCl_3_ administration to rats	Aβ accumulation, memory impairment, accumulation of phosphorylated tau, neuroinflammation, oxidative stress	^35^, ^36^
Streptozotocin administration to rats	Aβ accumulation and tau hyperphosphorylation in the hippocampus, neuroinflammation, oxidative stress, memory impairment	^37^
D‐gal administration to rats	Aβ accumulation, memory impairment, oxidative stress	^38^
LPS administration to rats	Aβ accumulation, neuroinflammation. memory impairment, neuronal death, oxidative stress	^39^
Aβ administration to mice/rats	Aβ accumulation, memory impairment	^40^, ^41^, ^42^
Tau administration to mice	Accumulation of phosphorylated tau, memory impairment	^43^, ^44^
***Selected primate models of AD***		
***Aging related***		
Old rhesus monkeys	Aβ accumulation, hyperphosphorylated tau, memory impairment, loss of myelin sheath, alterations in neurotransmission, degradation of synapses, activation of microglia and astrocytes	^45^, ^46^, ^47^, ^48^
Old cynomolgus macaques	Aβ accumulation, hyperphosphorylated tau, memory impairment, AMPA hyperactivation in the prefrontal cortex	^49^, ^50^, ^51^
Old mouse lemurs	Aβ accumulation, hyperphosphorylated tau, memory impairment	^52^, ^53^, ^54^
Induce Aβ in primates	Aβ accumulation, hyperphosphorylated tau, memory impairment, glial activation, and synaptic dysfunction	^55^, ^56^, ^57^
***Genetic***		
Tau mutation	Accumulation of phosphorylated tau, memory impairment, neuroinflammation, and neuronal degeneration	^58^
***Pharmacological***		
Formaldehyde administration	Aβ accumulation, hyperphosphorylated tau, memory impairment, neuron loss in the brain	^59^, ^60^
Streptozotocin administration	Aβ accumulation, hyperphosphorylated tau, memory impairment, neuronal degradation, and microglia activation	^61^, ^62^
Manganese administration	Aβ accumulation, memory impairment, and neuronal degeneration	^63^
***Selected zebrafish models of AD***		
***Genetic***		
*Psen1/Psen2* knockdown	Movement disorders	^64^
Human *APP* expression	Aβ deposition, neurodegeneration	^65^
Tau‐P301L line	Tau aggregation, neurodegeneration, and motor disorders	^66^
Tau‐152line	Tau aggregation and neurodegeneration	^67^
Tg‐line (eno 2: Tau)	Tau aggregation	^68^
***Pharmacological***		
Aβ administration to adult zebrafish	Aβ deposition, synaptic dysfunction, and impaired neurogenesis	^69^, ^70^
Aβ administration to embryos	Increased tau phosphorylation, cognitive impairment	^71^
Scopolamine administration	Memory impairment, increased anxiety, and CNS oxidative stress	^72^, ^73^
Okadaic acid administration	Aβ deposition, tau phosphorylation, cognitive impairment, induced oxidative stress, neuroinflammation, and dysfunction of the cholinergic system	^74^
Aluminum administration	Impaired motor activity and learning ability, increased acetylcholinesterase activity	^75^, ^76^
Manganese administration	Memory impairment, neuroapoptosis	^77^
Copper administration	Memory impairment, oxidative stress in brain, neuroinflammation	^78^, ^79^
Lead administration	Memory impairment	^80^

Abbreviations: Aβ, amyloid beta; AD, Alzheimer's disease; AMPA, α‐amino‐3‐hydroxy‐5‐methyl‐4‐isoxazolepropionic acid; CNS, central nervous system; LPS, lipopolysaccharide.

#### 2.1.1 Genetic rodent models

One of the major genetic approaches to modeling AD involves overexpressing the *APP* gene, leading to accumulation of Aβ peptides that aggregate into plaques. The *PDAPP* mouse model expresses three isoforms of *APP*, leading to excess Aβ and early formation of amyloid plaques, with spatial memory impairments at 6 months of age, mimicking early cognitive impairments of AD patients.^20^, ^81^ In a mouse Tg2576 model expressing the *APP695* isoform, Aβ plaques form by 11 months of age, paralleled by cognitive impairment from 10 months of age.^21^ A related model, the *APP23* strain, expresses the *APP751* isoform under the control of a neuron‐specific promoter, resulting in Aβ accumulation at 6, and severe cognitive impairment by 10, months of age.^22^ Although mouse strains like TASD or J20, combining multiple *APP* mutations, demonstrate more aggressive AD‐like pathology, these models still reflect primarily amyloid pathology rather than the full spectrum of AD (also including tauopathy, neuroinflammation, and synaptic dysfunction).^82^, ^83^ To complement mouse AD models, rat models include McGill‐R‐Thy1APP strain overexpressing *APP*, which causes Aβ deposition and hyperactivation of hippocampal neurons, more consistent with a clinical AD profile.^29^ Another important AD model involves mutations in *PSEN*, which encodes a component of the γ‐secretase complex responsible for cleaving APP into Aβ peptides.^84^ Mutations in *PSEN1* and *PSEN2* are linked to familial AD and lead to an increased production of the highly pathogenic Aβ42.^85^. However, *PSEN* mutations alone do not typically result in plaque formation, and are therefore often combined with *APP* mutations to enhance Aβ pathology. Many AD models incorporate the insertion of PSEN transgenes alongside APP mutations to create more robust models of the disease. For example, the PSAP and 2xKI mouse models, which harbor mutations in both *APP* and *PSEN* genes, exhibit accelerated Aβ plaque formation and enhanced pathological features.^86^, ^87^

**RESEARCH IN CONTEXT**

1. **Systematic review**: We reviewed existing literature on experimental animal models used to study the pathogenesis of Alzheimer's disease (AD), focusing on amyloid beta and tau accumulation, synaptic dysfunction, and neuroinflammation. Sources included peer‐reviewed studies on rodent, primate, and non‐mammalian models such as zebrafish, *Drosophila melanogaster*, and *Caenorhabditis elegans*.
2. **Interpretation**: No single animal model fully recapitulates the complex pathology of AD. However, a comparative, evolutionary biology approach provides critical insights into the strengths and limitations of different models. Our evaluation highlights the potential of non‐traditional models in complementing mammalian systems to enhance mechanistic understanding, improve preclinical translational value, and refine therapeutic screening strategies.
3. **Future directions**: While traditional animal models have significantly contributed to AD research, their translational limitations underscore the need for more physiologically relevant alternatives. Humanized rodent models, zebrafish, organoids, and induced pluripotent stem cell–based systems offer promising avenues, yet each has inherent drawbacks. A multimodal strategy integrating these models—coupled with advancements in bioengineering and genetic editing—may be crucial for bridging the gap between preclinical research and clinical application.

A prime example of a “combined” approach to AD modeling is the 5xFAD mouse strain that carries five mutations — three in *APP* and two in *PSEN* — and shows rapid AD‐like disease progression. Their Aβ accumulation begins as early as 1.5 months of age, followed by cognitive decline at 4 months and motor dysfunction and neuroinflammation shortly thereafter. This model has played an important role in studying not only Aβ deposition, but also neuroinflammation and axonal degeneration in AD.^24^, ^25^, ^26^ Similarly, the TgF344‐AD rat carrying mutations in *APP* and *PSEN1* exhibits amyloid deposition, cognitive decline, and widespread neuronal apoptosis, making it a valuable model for studying late stages of AD.^30^ Interestingly, despite the absence of mutations in the tau gene, hyperphosphorylation of tau is observed in TgF344‐AD rats, consistent with the endogenous tauopathy observed in AD patients, making this a reliable model to study the interaction between amyloid and tau pathologies.^30^, ^31^

Although amyloid pathology is a key AD feature, tauopathy — the accumulation of hyperphosphorylated tau in neurofibrillary tubules — also plays[Fig alz70273-fig-0001] a[Fig alz70273-fig-0002] critical role in neurodegeneration, and correlates more closely (than amyloid plaques) with cognitive decline.^88^ The 3xTg‐AD mouse model carrying mutations in *APP*, *PSEN1*, and tau is actively used to study the interaction between these proteins. Although Aβ accumulation and cognitive decline are observed by 6 months of age, tau pathology appears in only 50% of mice at this age, and all neuropathologic symptoms worsen with age.^27^ This model is particularly useful for studying the progression of tauopathy, which is crucial for understanding the later stages of AD. In addition to Aβ and tau, *APOE* is a significant risk factor for sAD. The *APOE* ε4 allele is associated with an increased risk of AD, while *APOE* ε2 is considered protective. The EFAD mouse line combining mutations in *APP* and *PSEN1* with human *APOE* isoforms provides a platform to study the effects of *APOE* on Aβ deposition, tauopathy, neuroinflammation, and synaptic function. Notably, murine *APOE* differs from human *APOE* by 100 to 300 amino acids, which affects Aβ accumulation and neuroinflammation, and may be critical for probing the impact of *APOE* variance on AD pathogenesis.^28^

Mounting evidence shows[Table alz70273-tbl-0001] that the *MICROTUBULE* *‐* *ASSOCIATED* *PROTEIN* *TAU* (*MAPT*) gene polymorphisms increase AD risk.^89^ Inclusion of pathogenic variants of human *MAPT* helps mimic the genetics of various human tauopathies. However, current tauopathy models rely on overexpression and may therefore contain artifacts. In contrast, the newest mouse models of tauopathy reproducibly display specific tau pathology using a knock‐in strategy. The effect of mutations of *MAPT* in hTau‐KI mice show positive phospho‐tau signals in the entorhinal cortex (4‐repeat tau expression patterns).^90^ A multigene approach is also used to create late‐onset AD (LOAD) mouse models (combining *APOE* ε4, TREM2 R47H, humanized Aβ42, and a high‐fat diet) to study the influence of environmental factors on disease progression. By 12 months of age, blood glucose and cholesterol levels are also elevated in these mice.^91^ The LOAD mice also display higher blood concentrations of proinflammatory cytokines tumor necrosis factor alpha (TNF‐α) and interleukin (IL)‐10, as well as neurofilament light chain,^91^ hence more accurately mimicking human tauopathies and AD.

For the past three decades, *APOE* has been known as the single greatest genetic modulator of sAD risk.^92^, ^93^ To systematically examine the role of *APOE* in age‐dependent neuroinflammation, mice expressing human *APOE* can be used, whose RNA sequencing reveals altered expression of glial *APOE* ε4, particularly in subsets of metabolically distinct microglial cells, during aging or inflammatory challenge.^92^ Mounting evidence links *APOE* to broader aging‐related brain changes, such as neuroinflammation, impaired energy metabolism, loss of myelin integrity, and increased blood–brain barrier (BBB) permeability.^94^ Novel mouse models and technological advances have also enabled a number of therapeutic approaches aimed at attenuating the *APOE* ε4‐linked sAD risks.

Likewise, obese *Ob/Ob* mutant mice (expressing inactive leptin) can also be used to model AD.^23^ Leptin inhibits the expression of *BACE*, the gene encoding a secretase that cleaves the Aβ precursor, whereas leptin contributes to the reduction of tau phosphorylation, neurogenesis, synaptogenesis, and risks of developing dementia.^23^ The *Ob/Ob* mice show anxious behavior, absence of long‐term potentiation in the hippocampus, and increased tau phosphorylation.^95^ A similar line of Db/Db mice expressing an inactive leptin receptor exist, showing impaired spatial learning and memory.^88^

#### 2.1.2 Transgenic models: limitations and transition to knock‐in models

Rodent models have been instrumental in advancing our understanding of AD pathogenesis, with transgenic mouse models historically dominating the field. Among these, the *5xFAD* and *APP/PS1* models^96^, ^97^ have been widely used due to their rapid amyloid plaque formation and cognitive impairments. However, these models rely on excitatory neuron‐specific promoters (e.g., Thy1, CamKII) to overexpress *APP* and *PSEN1*, limiting transgene expression to a subset of excitatory neurons. This approach stems from decades of AD research focused primarily on neuronal Aβ production. While excitatory neurons are indeed key contributors to Aβ deposition, emerging evidence suggests that such models may not fully recapitulate the complex, multicellular pathology of human AD.^98^

In particular, recent findings have shown that plaque burden in the *5xFAD* mouse model, and potentially in other transgenic lines using the Thy1 promoter, can vary significantly depending on whether the transgene is inherited maternally or paternally. Specifically, paternal inheritance has been associated with a markedly higher plaque load, likely due to genomic imprinting effects on promoter activity.^99^ This introduces an important source of variability in experimental outcomes. Notably, the majority of published studies using *5xFAD* mice do not report parental origin, underscoring the need to monitor and report breeding schemes stringently in AD research.

To address this limitation, the next generation of AD mouse models shifted toward knock‐in strategies, which avoid artificial overexpression of APP. For example, knock‐in AD mice created by replacing exons 16 and 17 of the murine App locus with humanized pathogenic sequences, induces physiologically relevant amyloid deposition without transgene overexpression.^100^ Such models, including *AMYLOID PRECURSOR PROTEIN with Swedish (NL), Arctic (G), and Iberian (F) Mutations* (*A* *PP* *NL* *‐* *G* *‐* *F*) and related mouse strains, better mimic familial AD pathology while preserving endogenous regulatory mechanisms. Unlike other transgenic models, knock‐in mice allow for a more nuanced investigation of Aβ dynamics across different cell types. Furthermore, recent findings underscore the importance of non‐neuronal Aβ production in AD. While traditional models emphasize excitatory neurons, gamma aminobutyric acid (GABA)‐ergic interneurons and oligodendrocytes also produce Aβ and contribute to plaque formation in vivo.^101^, ^102^ Thus, moving beyond a neuron‐centric perspective and considering the broader cellular landscape of AD pathogenesis are needed for future model development to ensure that experimental paradigms more accurately reflect the diverse contributions of different brain cell types to AD pathology.

#### 2.1.3 Pharmacological rodent models

Complementing genetic models, pharmacological rodent models of AD pathogenesis appear as a first‐intent choice model to rapidly screen the symptomatic or neuroprotective potencies of therapeutic strategies. Early studies applied the administration of aluminum chloride (evoking neurotoxicity due to its ability to accumulate in the brain) to induce AD‐like pathologies, including an increase in Aβ concentration, phosphorylated tau, neuroinflammation, cognitive decline, and heightened systemic oxidative stress, all of which contribute to memory dysfunction,^35^, ^36^ without assessing the formation of Aβ plaques. Some endogenous substances, such as Aβ peptides, have shown significant potential for generating more specific and translationally relevant AD‐like models in animals. Direct injection into the rodent brain of predominant Aβ forms, such as Aβ1‐40, Aβ1‐42, and shorter peptides (e.g., Aβ25‐35), generates an acute model that mimics sAD.^103^

The validity of the non‐transgenic model for AD physiopathology was established by assessing the extent and time course of toxicity induced by Aβ25‐35 injection and the differential vulnerability of brain structures. A single intracerebroventricular (i.c.v.) injection of aggregated Aβ25‐35 in rats induced short‐ and long‐term memory impairments and modified endogenous amyloid processing, with specific time‐course and regional responses.^104^ Various protocols have been applied for i.c.v. injections (into the lateral, dorsal third, and fourth ventricles) and intracerebral injections (into the hippocampus, hypothalamus, septum, median eminence, basal nucleus of Meynert, amygdala, frontal cortex, and cerebellum).^104^, ^105^, ^106^ After injection, Aβ localization and morphological alterations were assessed after 1, 2, and 3 weeks. The robustness of the i.c.v. model in introducing well‐characterized soluble Aβ species into the rat brain is supported histologically by fewer viable neurons, increased tau levels, and reduced dendritic spine density. Nineteen days after bilateral single i.c.v. administration of Aβ1‐42, proliferation of ependymal cells in the subventricular zone and atypical neuronal architecture in the hippocampus and cortex are also observed.^19^ Notably, neurons in the CA3 and dentate gyrus (DG) were more resistant to amyloid exposure.

A single i.c.v. injection of Aβ25‐35 induces structural changes not only in AD target structures, such as the cerebral cortex, nucleus basalis of Meynert, and CA1 region of the hippocampus,^107^ but also in the locus coeruleus.^108^ Injections into the entorhinal cortex resulted in Aβ deposition in the molecular layer of the dentate gyrus, while injections into the striatum induced Aβ deposition in the overlying neocortex, suggesting that pathology spreads along fiber tracts.^109^ The regional heterogeneity of lesions may stem from region‐specific intrinsic factors and the differential sensitivity of cell types.^110^ Early local changes gradually affect associated brain structures, leading to the spread of pathology. Therefore, understanding disease evolution in the context of time, cell types, and networks is essential for advancing AD research.

At high concentrations, Aβ forms oligomers and plaques that induce toxicity, inhibit synaptic transmission and plasticity, elevate intracellular calcium levels, and impair neurocircuitry function.^111^ For example, a single i.c.v. injection of Aβ25‐35 into the lateral brain ventricles induces synaptic dysfunction and structural damage, characterized by atypical size, shape, and branching architecture of neurons, in AD target brain structures, including the cerebral cortex, nucleus basalis of Meynert, and CA1 region of the hippocampus.^112^, ^113^ Additionally, the injection results in deficits in spatial memory and neurotransmitter imbalance. Notably, alongside this spatial memory deficit, altered levels of neuroactive amino acids such as glutamate and GABA, as well as taurine, glycine, and aspartate, have been observed.^19^, ^114^ Disturbances in the monoaminergic system, including dopamine, norepinephrine, and serotonin, have been reported.^115^ Furthermore, neural network dysfunction underlying memory impairments has also been observed in this model.^116^

Mounting evidence links Aβ25‐35 to aberrant APP misprocessing, tau system dysfunction, neuroinflammation, oxidative stress, cholinergic and glutamatergic deficits, synaptic deficiency, cell death, and cognitive decline.^117^ Endogenous Aβ1‐42 promotes Aβ aggregation, neuroinflammation, CNS oxidative stress,^103^ and excitotoxicity that disrupts synaptic plasticity and signaling.^118^ For instance, a single i.c.v. injection of Aβ1‐42 can alter acetylcholinesterase activity and modulate proinflammatory factors (e.g., TNF‐α, cytokines, IL‐6, IL‐1β, and brain derived neurotrophic factor).^119^ In vivo Aβ1‐42 injections also alter hippocampal long‐term potentiation and depression via partial astrocyte involvement,^120^ upregulate synaptic adenosine A2A receptors (A2AR),^121^ and enhance adult neurogenesis.^122^, [Fig alz70273-fig-0003]

Another interesting injectable AD model is the FAB model, involving ferrous, Aβ, and buthionine (FAB) components. Aβ1‐42 aggregation triggers neurodegeneration, while ferrous sulfate and buthionine sulfoximine induce oxidative stress by generating reactive oxygen species (ROS) and inhibiting the glutathione system.^123^, ^124^, ^125^ Likewise, i.c.v. injection of streptozotocin in rats causes Aβ deposition and tau hyperphosphorylation in the hippocampus, as well as neuroinflammation, oxidative stress, and cognitive impairments.^37^ Intraperitoneal administration of D‐galactose in rats leads to Aβ accumulation and memory impairments, which are exacerbated when combined with aluminum chloride.^38^, ^126^ Administration of lipopolysaccharide (LPS) induces neuroinflammation and pro‐inflammatory cytokine production (e.g., IL‐1β, IL‐6, TNF‐α, and transforming growth factor beta [TGF‐β]), enhancing amyloid precursor expression and β‐secretase activity, thereby promoting Aβ formation.^39^ Acute LPS administration results in Aβ accumulation, memory impairment, and neuronal death driven by elevated cyclooxygenase‐2 (COX‐2) and extracellular signal‐regulated kinase (ERK) activity,^39^ strongly linking oxidative stress through mitochondrial dysfunction, ROS, and nitric oxide (NO) production to AD pathogenesis.^39^ Importantly, all these pharmacological models share a common feature—the induction of oxidative stress, which emerges as an important target for studying synaptic dysfunction and cognitive decline in AD.^108^, ^127^, ^128^ Models involving intrahippocampal administration of oligomers of Aβ or tau proteins to rats or mice are also useful, helping study the acute effects of elevated levels of Aβ or tau on AD pathology and suggesting their distinct, independent detrimental effects on memory.^40^, ^41^, ^42^, ^43^, ^44^

Finally, aging‐based models have been used to study AD, as age is the most significant risk factor for the disease.^129^ Whole age‐related cognitive impairment, apoptosis, and elevated Aβ42 levels have been observed in mice and rats; these changes do not necessarily reflect specific AD pathology.^33^, ^34^ The OXYS rat, a model of accelerated aging, begins to accumulate Aβ by 12 months of age, although cognitive decline is not observed at this stage, emphasizing the need for cautious interpretation of aging models.^32^ Although aging models provide valuable insights into age‐related changes in the brain, they lack the specificity of genetic and pharmacologic models, which are crucial for studying the distinctive features of AD.

**FIGURE 3 alz70273-fig-0003:**
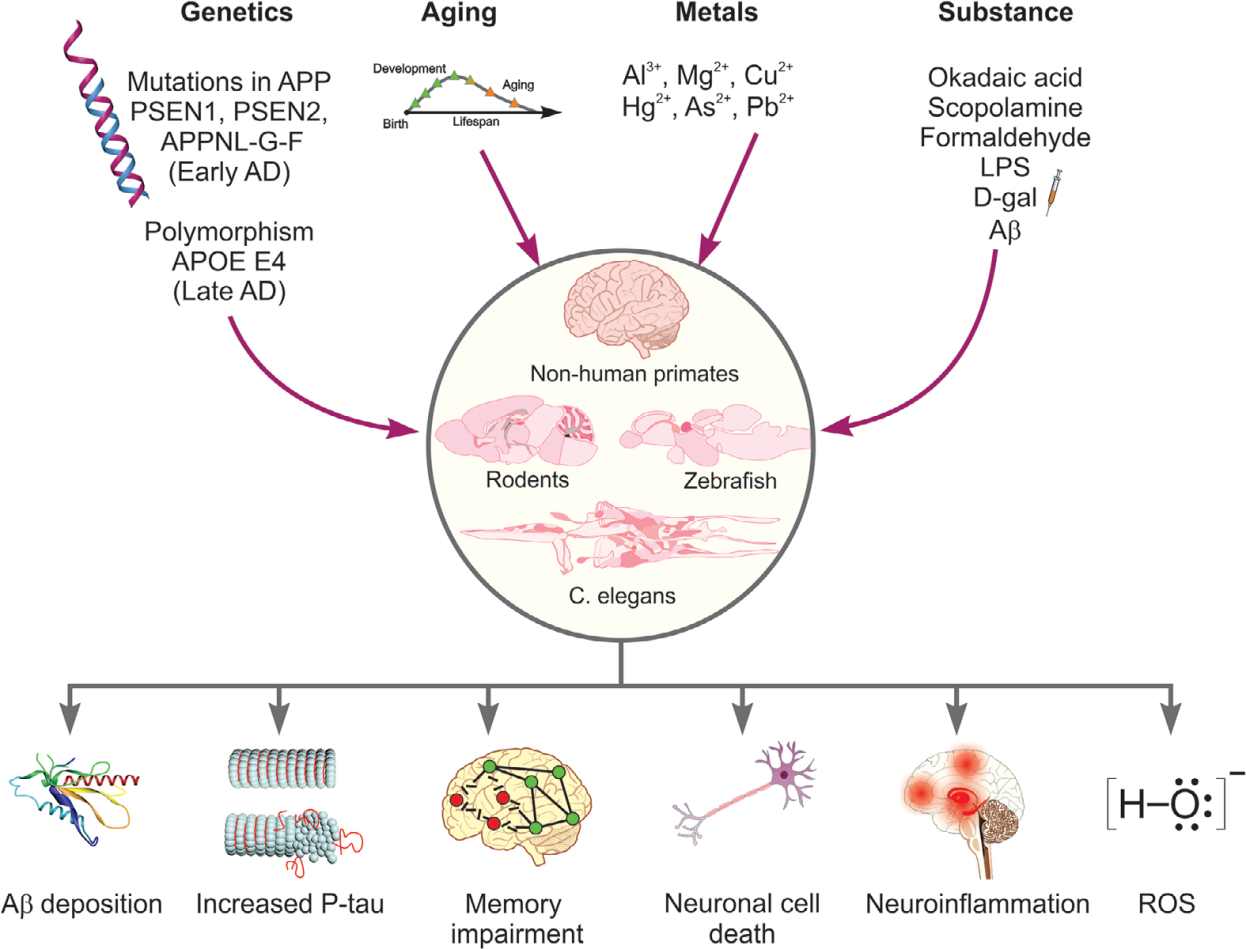
Summary of traditional approaches to modeling Alzheimer's disease (AD) in rodent models (top panel). Bottom panel illustrates common animal models used in AD research, categorized into genetic, aging, and injection models across species, such as mice, rats, non‐human primates, zebrafish, and invertebrates. Each cross‐point shows models (lines) applied in a specific group, with advantages of the animal models is provided. Aβ, amyloid beta; D‐gal, D‐ (+)‐galactose; LPS, lipopolysaccharide; p‐tau, phosphorylated tau; ROS, reactive oxygen species.

Overall, rodent genetic, pharmacologic and aging‐based models are indispensable tools for studying AD, with their own strengths and limitations, and targeting different aspects of the disease (Figure 3). As genetic models (e.g., 5xFAD, 3xTg‐AD, and EFAD) provide insight into amyloid and tau pathology, pharmacologic models offer a simpler but effective way to study the role of other factors (e.g., oxidative stress and neuroinflammation), whereas aging models, although somewhat less specific, further contribute to our understanding of AD and its progression.

### 2.2 Primate models of AD

The most commonly used primate species in neurodegenerative disease research are rhesus macaques (*Macaca mulatta*), blue‐eyed macaques (*Macaca fascicularis*), mouse lemurs (*Microcebus murinus*), and common marmosets (*Callithrix jacchus*;^130^ Table 1). Aging primates exhibit all major characteristic features of AD, including plaque deposition, tau pathology, and cognitive decline. In rhesus macaques, tau protein fibrillation begins in the entorhinal cortex at age 24 to 26 years and progresses with age, coinciding with the onset of cognitive impairment. Tau pathology spreads to the prefrontal cortex later in life, usually at 31 to 34 years, along with the appearance of Aβ plaques. Interestingly, phosphorylated tau can be detected in the entorhinal cortex as early as age 7 to 9 years, suggesting an early onset of tau pathology that mirrors the pattern seen in young adults clinically ^45^ Age‐related neurodegenerative changes in rhesus macaques also include degradation of synapses, especially glutamatergic axospinal synapses, and loss of myelin, while glutamatergic transmission is reduced without significant changes in cholinergic or GABA‐ergic systems.^46^, ^47^, ^48^ While neuroinflammation is manifested by activation of microglia and astrocytes, with Aβ oligomers also present, their deposits in macaques induce gliosis but not neurodegeneration or loss of synapses per se, which distinguishes them from human models, including both human cases and induced pluripotent stem cell (iPSC)‐derived models.^131^, ^132^, ^133^, ^134^ While not all aged macaques develop AD‐like pathologies, a subset of aging macaques can acquire Aβ deposition, tau pathology, and cognitive deficits.^135^ The fact that these pathologies can emerge in aging macaques, given their age‐related changes and brain structures more similar to those in humans than rodents, makes them a valuable model for studying AD. This variability reflects the complexity of the disease and underscores the relevance of aged primates as models that can potentially best recapitulate human AD.

Cynomolgus macaques develop tau pathology and accumulation of Aβ starting at ≈ 20 years of age.^49^, ^50^ Notably, in this model, hyperactivation of adenosine monophosphate (AMP)‐activated protein kinase (AMPK) in the prefrontal cortex contributes to the pathogenesis of AD. As AMPK inhibition alleviates cognitive impairment and synaptic dysfunction in mouse models, decreased postsynaptic density zone size in aging macaques is indicative of synaptic impairment.^51^ Mouse lemurs, albeit exhibiting tau pathology and deposition of Aβ plaques, show the latter accumulation only in individuals 7+ years of age, with tau pathology found in young animals.^52^ In contrast, common marmosets show Aβ deposition from ≈ 7 years of age, with a distribution similar to that observed in the human brain, but generally do not exhibit hyperphosphorylated tau, and only a small percentage survive to this age.^53^, ^54^ Finally, as diets high in saturated fat, sugar, and sodium—known risk factors for AD and obesity, diabetes, and cardiovascular disease clinically—can exacerbate neurodegenerative processes in primate models. For example, female macaques fed a Western diet showed decreased white matter volume, an early marker of AD pathology.^136^

Similar to rodent models, primates have also been used to administer Aβ to the brain. For example, i.c.v. injections of soluble amyloid oligomers into the lateral ventricle of cynomolgus macaques induce Aβ accumulation in brain regions associated with cognitive function, resulting in tau hyperphosphorylation, neurofibrillary tangle formation, glial activation, and synaptic dysfunction.^137^ This model has the advantage of minimal age‐related differences in outcomes, as animals of different ages show comparable results.^55^ In rhesus macaques, i.c.v. injections of soluble Aβ to adult, but not aged, females induces synaptic failure and neuroinflammation, but not tau hyperphosphorylation.^56^ In contrast, administration of insoluble Aβ to the brains of aged rhesus macaques increases such phosphorylation and induces neuronal loss.^57^ By comparing the pathological features of naturally aged and induced AD in non‐human primate (NHP) models, researchers have highlighted the importance of factors such as injection routes and frequencies for standardizing NHP models of AD. Notably, tissue and BBB damage caused by injection models can lead to the infiltration of peripheral immune cells, which does not occur in clinical AD. Induced NHP models can be generated through injections of Aβ oligomers, brain homogenates, neurotoxins, or formaldehyde. Intracerebroventricular delivery of Aβ not only induces proteopathies, but also activates innate immunity and allows immune cell penetration through the damaged BBB.^138^ Therefore, establishing clear guidelines is essential to enhance the translational potential of NHP models for clinical applications.

Notably, in some primate models, it is possible to achieve changes in tau levels without altering Aβ levels. For example, adult female rhesus macaques injected with an adeno‐associated virus expressing a tau double mutation in the entorhinal cortex exhibit tau pathology, neuroinflammation, and neuronal degeneration.^58^ Such results expand our understanding of AD and lay the groundwork for testing tau‐based therapeutics for AD patients.

Pharmacologic models of AD in primates include administration of formaldehyde, streptozotocin, and manganese. Chronic intravenous administration of formaldehyde to young rhesus macaques resulted in the development of amyloid plaques, neurofibrillary tubules, hyperphosphorylated tau, and cognitive impairment accompanied by neuronal loss.^59^ Chronic ingestion of 3% methanol, a metabolite of formaldehyde, can similarly cause cognitive decline, increased tau phosphorylation, and accumulation of amyloid plaques.^60^ Streptozotocin triggers AD‐like pathology by disrupting insulin signaling, leading to phosphorylation of the insulin receptor and impairment of downstream pathways. In blue‐footed macaques (*Mandrillus sphinx*), this triggers amyloid plaque formation, hyperphosphorylated tau, neuronal degradation, and microglia activation.^61^, ^62^ Chronic manganese exposure in primates has been associated with cognitive dysfunction and neurodegeneration. Exposure to manganese increases *APP* gene expression, leading to the accumulation of amyloid plaques and neuronal damage.^63^

Overall, primate models provide valuable and specific insights into the pathogenesis of AD, offering promising platforms to explore potential therapeutic interventions (Table 1) as species much closer to humans, both genetically and anatomically, thus improving AD research and preclinical anti‐AD drug testing. However, the creation and use of genetically modified NHPs present several challenges, both technical and ethical, which contribute to the relative rarity of such models in AD studies. These challenges can be categorized into five main areas: the welfare impacts of genetic engineering procedures, mother–infant separation, the genetic modification itself, experimental procedures, and laboratory housing conditions.^139^ These factors complicate the use of genetically altered primates in research and make them difficult to replace with genetically modified mice or other species. Moreover, current gene‐editing techniques, such as CRISPR/Cas9, are not without limitations. For instance, a significant concern is the risk of off‐target effects, where mutations may occur at unintended sites, leading to unforeseen phenotypes. These unintended genetic modifications can undermine the reliability of studies, as poorly designed experiments with insufficient controls or presentations may ultimately limit their translational potential.

### 2.3 Zebrafish models of AD

The zebrafish (*Danio rerio*) is a powerful model system in CNS research due to its genetic accessibility and well‐characterized genome with high (> 70%) homology to humans.^140^, ^141^ Zebrafish models provide valuable insights into the genetic, cellular, and behavioral aspects of AD pathology (Table 1). For example, zebrafish expressing human tau^142^ in neurons display hyperphosphorylation, cytoskeletal disruptions, and accumulation in neuronal cell bodies, mimicking human AD.^143^ Zebrafish strains (e.g., the Tau‐P301L fish) with mutations in the tau gene (primarily associated with frontotemporal dementia), also exhibit tau hyperphosphorylation, aggregation, neurodegeneration, and motor deficits.^66^ Although the Tau‐P301L mutation is mainly linked to frontotemporal dementia, these pathological features share common mechanisms with tau‐related changes observed in AD clinically. Other zebrafish models with tau gene mutations, such as the Tau‐152T strain, also show high levels of tau phosphorylation and neurodegeneration.^67^ Additionally, the Tg(eno2) model, which expresses mutant tau under the control of the enolase‐2 promoter, shows progressive tau accumulation with age, effectively mimicking in zebrafish the age‐dependent progression of AD.^68^ Collectively, these fish models have significantly deepened our understanding of tau aggregation and its role in AD neurodegeneration.

Zebrafish models have also contributed to our understanding of the role of Aβ aggregates in AD pathology. For example, Aβ injections into the fish brain ventricle led to Aβ aggregation, tau phosphorylation, and neurotoxicity, causing synaptic dysfunction and impaired neurogenesis, especially in older animals.^69^, ^70^ Furthermore, when Aβ is administered to embryos, fish cognitive abilities are also impaired, accompanied by increased levels of phosphorylated tau.^71^ Interestingly, Aβ overexpression in zebrafish increases neural cell proliferation and neurogenesis in the adult brain, a phenomenon unusual in human AD.^69^, ^70^

Zebrafish possess two orthologs of the human *APP* gene —*APPA* and *APPB* — with the latter playing a crucial role in the brain,^144^ as its knockout impairs cell adhesion and evokes neural maldevelopment.^145^ Transgenic zebrafish strains expressing human *APP* display Aβ deposition and neurodegeneration, providing another useful genetic model for studying Aβ pathology.^65^ As *PSEN* is strongly implicated in familial forms of AD,^146^ and zebrafish have orthologs of human *PSEN1* and *PSEN2*, fish models with *PSEN* *1* knockout exhibit impaired development of the histaminergic system, which is crucial for cognitive functions, as well as overt motor impairments.^64^ *PSEN2* knockout in zebrafish leads to neuronal death, mimicking neurodegeneration observed in clinical AD,^147^ and offers insights into the distinct roles of *PSEN* genes in AD, including their contribution to APP cleavage via γ‐secretase. However, it is important to note that the pathologies arising from psen deletion may not solely reflect AD‐related mechanisms. Given that presenilins are involved in the cleavage of numerous substrates critical for neuronal development, the observed phenotypes could also result from disrupted developmental processes independent of APP cleavage. This highlights the complexity of using *PSEN* knockout models to study AD and suggests that further research is needed to disentangle the specific contributions of PSEN function to AD‐like pathology.

Zebrafish are also extensively used in pharmacological models of AD. For example, scopolamine, a muscarinic acetylcholine receptor antagonist, is frequently used to model cognitive deficits, oxidative stress, and anxiety‐like behavior in zebrafish.^72^, ^73^ Additionally, okadaic acid, an inhibitor of protein phosphatases PP1 and PP2A, is used to study tau pathology in these fish due to its ability to induce tau hyperphosphorylation and cognitive deficits associated with AD.^74^ Okadaic acid models show increased tau phosphorylation, Aβ aggregation, and pGSK3 a/β, providing a pharmacological platform for studying AD‐like states in zebrafish. However, while okadaic acid also induces neuroinflammation and cholinergic dysfunction in other species, these changes have not yet been confirmed in zebrafish.^148^

Exposure to heavy metals, such as aluminum, manganese, copper, and lead, is associated with neurodegeneration and cognitive impairments in zebrafish (Table 1). Exposure to aluminum chloride in zebrafish larvae leads to glial cell damage due to decreased levels of glial fibrillary acidic protein (GFAP).^149^ In adult zebrafish, aluminum chloride causes impaired motor activity and learning ability, with these behavioral effects accompanied by increased acetylcholinesterase activity.^75^, ^76^ Notably, the observed decrease in GFAP expression in zebrafish contrasts with the increased GFAP levels typically seen in human AD and mammalian models, which is associated with reactive gliosis. This cross‐taxon discrepancy is interesting and may reflect species‐specific differences in glial cell responses or distinct mechanisms of aluminum‐induced neurotoxicity in zebrafish. It is possible that aluminum chloride exposure leads to glial cell damage and downregulation of GFAP expression rather than triggering a gliotic response, highlighting the need for further investigation into glial cell dynamics in this model.

Similarly, manganese chloride exposure leads to memory impairment and increased apoptosis in the zebrafish nervous system,^77^ likely due to its effects on the central cholinergic system,^150^, ^151^ albeit yet to be confirmed in zebrafish. Copper toxicity in zebrafish induces oxidative stress, neuroinflammation,^78^ and memory impairments.^79^ Lead is another well‐known neurotoxic agent, whose primary effect involves the generation of ROS and oxidative stress.^152^ Paralleling its clinical cognitive impairment effects in children,^153^ lead induces pronounced learning deficits in zebrafish that persist across generations.^80^ Thus, zebrafish are a promising model for modeling environmentally relevant neurodegenerative diseases, including AD, induced by heavy metal neurotoxicity.

Overall, zebrafish models provide a valuable platform for studying molecular and cellular mechanisms of AD. Furthermore, zebrafish offer logistical and technical advantages for research, including transparent embryos, a rapid development cycle, and suitability for large‐scale screening of drug effects, making them an efficient and versatile model system.^154^ Due to their small size (< 1 mm), zebrafish embryos and larvae can be easily placed in 96‐well plates, allowing for high‐throughput drug screening in a short period of time^155^ and demonstrating unique potential for screening novel anti‐AD drugs in complex in vivo vertebrate systems. Zebrafish can also model and track cognitive decline (as has been shown for treatment with 1‐methyl‐4‐phenyl‐1,2,3,6‐tetrahydropyridine [MPTP]^130^ in a different, Parkinson's model) in its dynamics using artificial intelligence (AI). AI has recently been increasingly used to analyze animal behavior, highlighting subtle phenotypic differences under the influence of drugs or as a result of CNS pathology,^156^, ^157^, ^158^, ^159^ thereby markedly expanding our knowledge of diseases and treatments, including in zebrafish models.

### 2.4 Invertebrate models of AD

Complementing vertebrate species, the use of invertebrates as model organisms offers several key advantages for AD research, including their rapid generation, analysis of transgenic lines, and high throughput. The most commonly used species for this include *Drosophila melanogaster* (the fruit fly) and *Caenorhabditis elegans* (a nematode, Table 2), particularly valuable due to its ease of cultivation and maintenance. Despite having only 302 neurons, *C. elegans* exhibits complex behavior, including social interaction and learning^160^, ^161^, ^162^ and uses multiple conventional neurotransmitters, making this roundworm a reliable model for studying conserved mechanisms of nervous system function.^162^, ^163^, ^164^, ^165^ In addition, for both *C. elegans* and *Drosophila*, complete connectomes comprising 302 and 139,255 neurons, respectively, have been mapped.^166^, ^167^ Representing the only animals for which all neuronal connections have been fully characterized, this greatly increases their value for the study of the pathogenesis of neurological diseases and the development of neurobiology in general.

**TABLE 2:** Selected invertebrate models of AD.

Model	Evoked abnormalities	References
***C. elegans***		
CL2006/GMC101 line	Aβ production, paralysis, systemic oxidative stress	^168^, ^169^, ^170^, ^171^
CL4176 line	Aβ deposition, cognitive impairment	^172^
Aβ1‐42 and tau expression	Aβ deposition, tau aggregation, impaired cognitive function, neuronal degeneration, and reduced longevity	^173^
Human tau expression	Impaired coordination of movements, accumulation of phosphorylated tau, and neurodegeneration	^174^, ^175^
Mutated human tau	Tau aggregation, paralysis, and acute neuronal dysfunction	^176^
***Drosophila***		
Human Aβ42 expression	Aβ deposition, memory impairment, and neurodegeneration	^177^
Human *APP* and *BASE* expression	Aβ deposition, impaired cognitive ability, and shortened lifespan	^178^, ^179^, ^180^
Tau expression	Tau aggregation, memory deficits, neuronal degeneration, and reduced lifespan	^181^, ^182^

Abbreviations: Aβ, amyloid beta; AD, Alzheimer's disease.

In *C. elegans, APL‐1* serves as an ortholog of the human *APP* gene, although this species does not produce Aβ peptides due to differences in amino acid sequence and lack of β‐secretase. To overcome this problem, the transgenic line CL2006 was generated, in which Aβ is produced in muscle cells, leading to paralysis and death.^168^ In addition, Aβ raises the levels of iron content and oxidative stress in SH‐SY5Y cells overexpressing the Swedish mutant form of human β‐amyloid precursor protein (APPsw) and in *C. elegans* Aβ‐expressing strain CL2006.^169^, ^170^ The onset of locomotor paralysis in these nematodes can be accelerated by inactivation of *epi‐1* encoding the α‐chain of laminin.^183^ However, Aβ produced in this line is a shortened fragment,^184^ unlike in clinical AD. In contrast, the GMC101 line produces full‐length Aβ‐42, which results in faster progression of paralysis.^171^ Temperature‐dependent control of Aβ‐42 expression is possible in lines such as CL4176, in which cognitive impairment and intraneuronal Aβ accumulation have been observed.^172^ Improved AD models in *C. elegans* are obtained by crossing Aβ1‐42 (line CL2355) with a transgenic strain expressing pan‐neuronal tau, either pro‐ (line BR5270) or anti‐aggregating tau (line BR5271).^173^ These models combine Aβ1‐42 expression with tau protein expression, resulting in cognitive impairment, neuronal degeneration, and shortened lifespan,^173^ as well as hyper‐expression of human *APOE* alleles. Co‐expression of *APOE* ε4 and human *APP* in *C. elegans* further exacerbates neurodegeneration in an *APP*‐dependent manner.^185^

Several models of *C. elegans* target tauopathies, based on the expression of human tau in neurons that leads to impaired movement coordination, accumulation of phosphorylated tau, and neurodegeneration. A line carrying the *FTDP‐17* mutation (associated with frontotemporal dementia clinically) exhibits similar but more severe AD‐like phenotypes.^174^, ^175^ In addition, a model expressing tau with the *A152T* mutation (found in some tauopathies clinically) demonstrates acute neuronal dysfunction and paralysis, although only soluble tau oligomers accumulate in these worms.^176^ In other models, expression of pseudohyperphosphorylated tau is observed, resulting in developmental defects in motor neurons.^186^

In *Drosophila*, modeling neurodegenerative diseases usually involves adult flies, when the fly brain contains associative areas consisting of ≈ 2500 neurons critical for olfactory learning and memory.^187^ Like *C. elegans*, wild‐type *Drosophila* does not produce toxic Aβ in vivo. A transgenic line expressing human Aβ42 in the nervous system was created to model AD, which leads to age‐related memory impairment and neurodegeneration^177^ that may be linked to slowed neurotransmission due to Aβ accumulation.^188^

Another approach in *Drosophila* AD models involves expression of human *APP* in combination with human *BACE1* (*APP*/*BACE*), resulting in the release of *APP*, which is cleaved by human *BACE1* and endogenous γ‐secretase. Such flies exhibit Aβ deposition, cognitive decline, and shortened lifespan, as well as synaptic dysfunction, impaired motor activity, and reduced number of neuromuscular synapse boutons.^178^, ^179^, ^180^, ^189^ Co‐expression of human tau further enhances neurodegeneration,^135^ and while tau expression alone also causes memory deficits, neuronal degeneration, and shortened lifespan, mutant tau causes more severe pathology.^181^, ^182^

## 3 DISCUSSION

**FIGURE 4:**
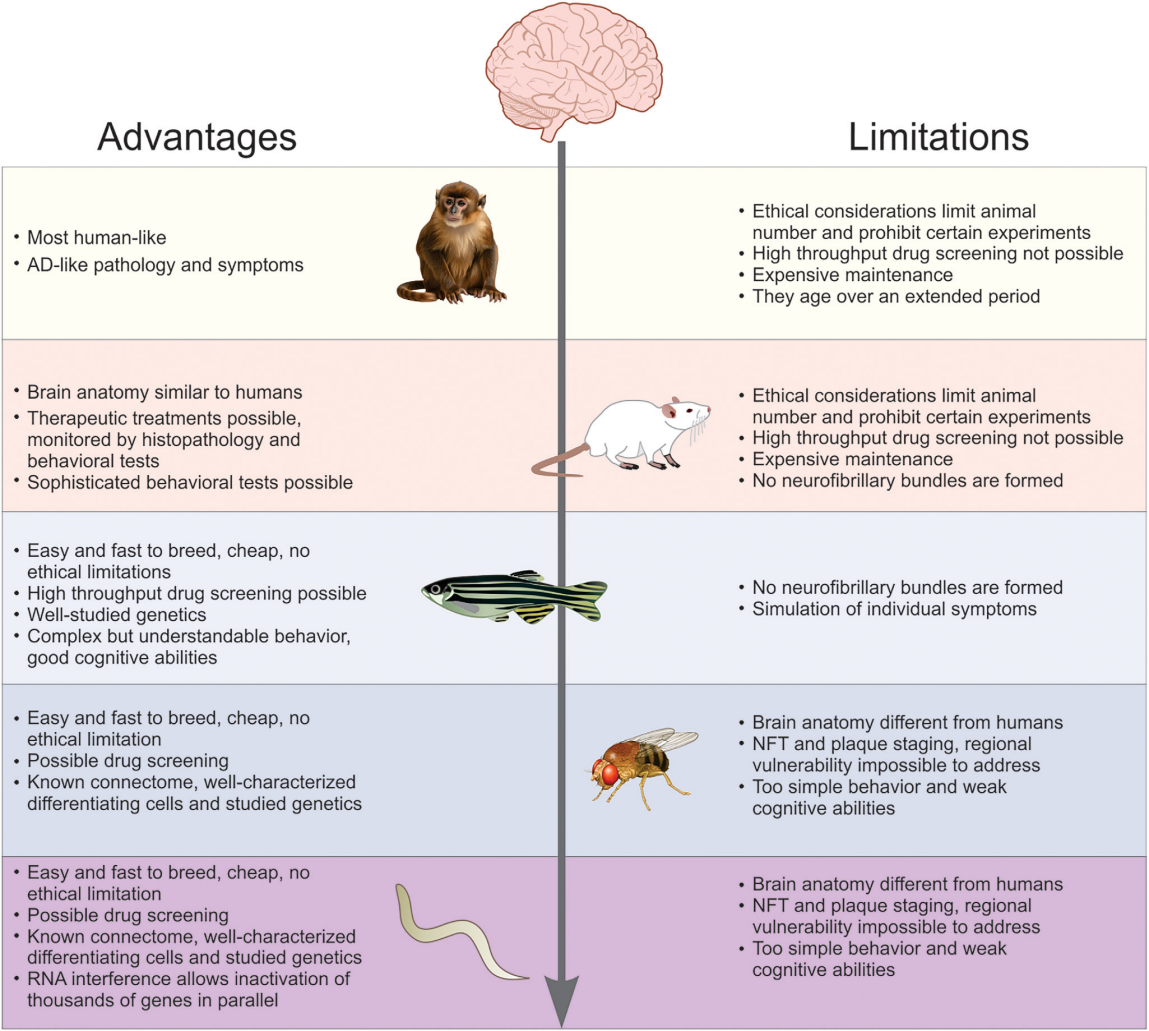
Comparative analyses of major model organisms used in AD research, including primates, rodents, fish, *Drosophila melanogaster*, and *Caenorhabditis elegans*, highlighting their main advantages and limitation for AD modeling. AD, Alzheimer's disease; NFT, neurofibrillary tangle.

Comparative analyses of major model organisms used in AD research are briefly summarized in Figure 4. While translational AD research relies heavily on a wide range of animal models, a logical question that arises is whether such variety is necessary, and why? On the one hand, rodents, particularly transgenic mice, are most commonly used to study AD, especially its familial forms and some aspects of sAD (Table 1). Yet, because sAD is more common in humans, these models may not fully capture the complexity of the disease, and also produce different isoforms of Aβ, some of which are non‐toxic (e.g., Aβ_1–38_ ^190^), further complicating direct interpretation of the results across species. Transgenic rats, such as the TgF344‐AD strain, represent a more accurate model of AD pathogenesis, and exhibit elevated levels of toxic Aβ peptides (Aβ 1‐40, Aβ 1‐42) and tauopathy without a mutated tau gene, hence better mimicking human AD pathology than mice.^30^ Rats also express six human tau isoforms (vs. only three in mice), hence allowing for a more accurate study of tauopathies.

Furthermore, sex differences are also crucial to consider in AD modeling. For instance, female *APP/PSEN1* transgenic mice exhibit more rapid cognitive decline than males, reflecting a higher prevalence of AD in females, making them useful for studying sex differences.^191^, ^192^ Notably, female *5xFAD*,^193^, *APPPS1*,^194^ and *APPNL‐G‐F* ^101^ mouse models show a higher Aβ burden and more severe cognitive deficits compared to males, further emphasizing the role of sex as a biological variable. On the other hand, non‐transgenic models that inject tau or Aβ, while mimicking elevated levels of these proteins and their independent effects on the brain, often fail to reproduce the gradual progression of AD observed in humans.^124^ In addition, sex dimorphism has been reported in primate models, in which female aged rhesus macaques exhibit more pronounced tau pathology and cognitive decline compared to males.^195^ Thus, because both transgenic and non‐transgenic models cannot capture the full spectrum of AD symptoms, considering sex‐specific differences and applying a combined approach using multiple models and a wider range of species may be warranted.

The potential role of physical activity in the treatment of AD is another area in which rodent models excel. For instance, studies using physical activity protocols in rodents help examine how exercise in combination with pharmacologic agents affects AD progression. Although these factors have been studied separately, their combined effects remain poorly understood.^196^, ^197^, ^198^, ^199^ Other lifestyle factors, such as diet and stress, also influence the development of AD, and studying their combination in animal models may lead to the development of new treatment strategies (Table 2). The impact of environmental enrichment, highly beneficial for neuroplasticity in general,^200^, ^201^ also can be rigorously used in studies focusing on AD therapies. Detrimental environmental exposures, particularly toxins, represent another important aspect of AD research. While toxins are recognized as risk factors for the development of AD,^202^ laboratory animals are usually kept in a controlled environment free from such exposure, raising questions about how significant environmental toxins are for AD pathology and whether these factors should be considered in research using its animal models.^203^ Thus, although rodents are widely used in modeling various neurodegenerative diseases, including AD, they have certain limitations (Figure 3), including differences in microglial activation and immune responses that are different from humans, which may affect the study of neuroinflammation. Likewise, their cortical organization is different from humans, which does not allow for a full modeling of cognitive impairment in AD.

The incomparable advantage of using primates is their evolutionary kinship with humans, which allows more accurate modeling of AD pathology per se. For example, signs of pathology in monkeys are observed mainly in the prefrontal cortex, as in humans, whereas in rodents AD‐like pathology is found mainly in the hippocampus.^51^ In primates, AD pathology is related to glutamatergic synapses in the prefrontal cortex, because they are critical for cognitive function, and are more abundant in monkeys versus rodents, which may also influence AD‐like pathologic trajectories in the two model groups.^45^ At the same time, species differences must also be considered, given the presence of neurogenesis in macaques and mice, but much less in humans (mainly in the dentate gyrus and olfactory bulb).^203^ This aspect is important because overt neurogenesis may prevent the development of neurodegeneration in AD models, thereby limiting their utility. Furthermore, the primary model of AD‐like neurodegeneration in primates is aging, and pharmacologic models require adult animals. Because of their longevity, such models require large time resources as well as large maintenance resources, making it difficult to use primates as model subjects. Likewise, large‐scale AD drug research in mammals (especially in primates) is costly and limited by strict ethical regulations due to closeness to humans, whereas genetic engineering in primates is much more difficult and less common compared to rodents.

As alternative non‐primate and non‐rodent models, *TAU* *‐* *P301L* dogs and cats have also been shown to develop AD‐like pathologies, including amyloid deposition and cognitive decline. Although modeling AD in these species remains relatively unconventional, their longer lifespan and naturally occurring pathologies offer unique advantages for studying late‐stage disease progression. However, the use of these models is limited by genetic variability and ethical considerations. Therefore, contrasting the potential of such models with the more commonly used rodent and primate models provides valuable insight into the translational relevance and limitations of each approach.

While in terms of practicality zebrafish represent by far more affordable and efficient alternatives for translational AD research, these fish also have some clear limitations. For example, they regenerate neurons throughout life, which complicates AD research because such regeneration prevents neurodegeneration.^204^ Nevertheless, this ability to regenerate neurons in zebrafish may be useful for studying mechanisms of toxin resistance, which has implications for understanding the pathogenesis of AD.^204^ While zebrafish are not ideal for testing therapeutic agents because of their inherent active neurogenesis,^204^ this aspect may make them more resistant to various toxic agents, hence enabling a better focus on the mechanisms of resistance to AD pathology. In addition, zebrafish also present limitations for being an acortical animal.^205^, ^206^ For example, studies have shown that Aβ accumulates in the cortex,^207^ a process that cannot be precisely replicated in zebrafish.

Furthermore, zebrafish have a much simpler nervous system and cognitive repertoire compared to mammals, which limits their usefulness for studying complex cognitive disorders. However, mounting recent evidence continues to unravel the developing complexity zebrafish behavior^208^ and cognitive phenotypes,^209^ especially empowered by AI‐driven approaches,^156^, ^157^, ^158^, ^210^, ^211^ hence suggesting higher potential utility of zebrafish for studying AD‐like symptoms than it is presently recognized. Due to their overly simpler nervous systems, invertebrate model objects, such as *Drosophila* and *C. elegans*, are presently used to study only some aspects of AD. Their clear value is the simplicity of maintenance and rapidity of research, as well as the possibility of creating transgenic models.^212^ For example, *C. elegans* can be used as an effective screen to assess the impact of test treatments on amyloid deposition removal.^213^ However, there are also significant differences between the APP and tau proteins of these organisms and humans that must be considered,^214^ especially because both invertebrate models lack amyloid plaques and tau bundles.^215^

In summary, current state of the art of AD modeling resembles the famous *The Wizard of Oz* novel, in which each main hero lacks a unique, but critically important, trait. For example, while rodents are generally well suited to study AD‐like behavior and genetic and pharmacological manipulations, they are limited by anatomical differences and inability to fully model tau pathology. While rodents do not develop Aβ plaques during aging (unlike humans), due to differences in Aβ sequences between species,^216^ advances in genetic research and disease modeling have led to the development of more comprehensive models that have clarified the causal relationships between Aβ and AD.^217^ The inability of current AD models to fully mimic the pathology leads to inconsistent results between preclinical and clinical phases. Primates are closest to humans in brain structure and function, but are expensive, slow to develop AD, and ethically challenging. Zebrafish are highly efficient for genetic, drug screening studies and imaging, but neurogenesis complicates neurodegeneration studies. *Drosophila* is a fast, cost‐effective model for genetic and molecular studies, but has limited relevance to cognitive decline, and does not display major hallmarks of this pathogenesis. *C. elegans* is an ideal model for simple genetic studies, but not sophisticated enough to model progressive AD pathology and behavior (Figure 3). Their short lifespan may also limit studying progressive aspect of AD, especially critical for sAD. Future directions may involve developing non‐genetic models of sAD, especially in organisms with shorter life spans, such as *Drosophila* and *C. elegans*, and identifying early biomarkers that may be translated to other species, allowing earlier therapeutic interventions. Thus, a more complete understanding of AD may require systematic integration of knowledge from multiple models, in parallel with novel analyses of their common and different molecular (e.g., genomic, proteomic, and metabolomic) profiles associated with AD.

AD pathogenesis is linked to neuroinflammation, regulated by neuroglial cells, including microglia and astrocytes that are both increasingly implicated in this disorder. For example, activated microglia is present at sites of Aβ deposition,^218^, ^219^ mutations of the microglia receptor TREM2 result in hyperphosphorylation of tau,^220^ whereas knockout of *CR3*, the complement receptor 3, in *APP*‐transgenic mice lowers Aβ deposition.^221^ In addition, microglia can interact with Aβ and *APP*, contributing to the induction of amyloid peptide clearance from the brain.^222^, ^223^, ^224^ Overall, microglia play a dual role in AD: its moderate activation provides a protective function, while its over‐activation induces an inflammatory response that can exacerbate neurodegeneration.^225^, ^226^ Astrocytes, like microglia, are capable of both protective function (by lowering Aβ) and promoting the inflammatory response^226^, ^227^ that can lead to neurodegeneration.^228^, ^229^, ^230^, ^231^, ^232^ Although the role of microglia in amyloid plaque formation remains controversial, emerging evidence highlights their critical involvement in Aβ pathology. In transgenic knock‐in mouse models of Aβ amyloidosis, apoE aggregates within the microglial endolysosomal system acting as seeds for Aβ plaque formation.^233^ Moreover, depletion of microglia impairs plaque seeding, further supporting their essential role in Aβ deposition.^234^ These findings add more depth to the dual role of microglia in AD pathogenesis, where they not only contribute to inflammatory cascades but also directly participate in Aβ plaque dynamics through apoE‐dependent mechanisms.^233^ This underscores the need for more nuanced studies on microglial function in AD models across species. Thus, a possible strategy for treatment may not be to suppress or activate glia, but rather to fine‐tune the regulation of these cells, necessitating more nuanced studies of glial function in both traditional and novel model organisms. For example, in *C. elegans*, glia are not required to maintain neuronal activity,^235^ which is interesting in the context of AD modeling, helping to distinguish between primary and secondary effects of glial manipulations on neuronal activity. However, studying the role of microglia in AD should also consider cross‐taxon differences. While human glia exhibit considerable heterogeneity across various subtypes, reactive states of microglia, astrocytes, and oligodendrocytes are also present in other species, including mice, macaques, and monkeys, particularly in the context of AD.^236^, ^237^, ^238^ Additionally, in humans and rodents, a large number of differentially expressed genes, including genes associated with neurodegenerative diseases, are detected in microglia. At the same time, the highest similarity in the expression of genes associated with AD has been shown in macaques,^239^ likely representing the closest model object for studying the role of human microglia in AD.

Impaired autophagy leads to accumulation of Aβ peptides and is also a hallmark of AD.^240^ Because autophagy is closely related to the functioning of lysosomes, providing destruction of macromolecules, lysosomal dysfunction also contributes to AD, in particular, the endosomal–lysosomal pathway is the site of APP cleavage, as well as tau hyperphosphorylation.^241^ Furthermore, autophagy can be regulated by mammalian target of rapamycin (mTOR), as reducing its hyperactivity in AD mice reverses cognitive deficits and repairs CNS autophagy.^242^, ^243^, ^244^ Rodent models may be the best option to study the features of mTOR regulation in AD, as the structural and functional elements of the mTOR system show better affinity with that of humans versus zebrafish and flies.^245^

The role of Aβ in AD pathogenesis is currently reconsidered,^246^ especially because almost all therapeutic agents with good results in animal models have no therapeutic effects clinically.^247^ One possibility can be that Aβ accumulation — the buildup of Aβ in various forms, including soluble Aβ, oligomeric species, or amyloid plaque deposition — For example, studies have shown that Aβ accumulates in the cortexmay be the result of a protective response rather than the cause of the pathology, and therefore amyloid peptide treatment would not produce the desired results, hence leading us to question the relevance of models based on *APP* overexpression or Aβ administration^248^ and calling for principally new models of AD. However, the failure of anti‐AD drug candidates in clinical trials may be due to the fact that therapy was started too late, that is, when overt and incurable neurodegeneration has already developed. It is important to note that the failure of therapeutic strategies aimed at combating Aβ is likely due to the threshold concentration of Aβ. For Aβ deposition in vivo, a critical concentration is required. In the early stage, plaque density increases rapidly, after which it stabilizes as the disease progresses.^101^, ^249^ Aβ plaque deposition follows sigmoidal kinetics, meaning that once this threshold concentration is reached, amyloidosis, including plaque deposition, occurs.^249^ The late intervention of various drugs in human patients could thus miss the onset of this threshold concentration, after which anti‐Aβ therapeutic strategies have very limited effects. This, in turn, raises the question of improving diagnosis and finding new markers for the disease. In general, the problem of finding early markers of AD is highly relevant, as this would make it possible to apply therapy before the stage of neurodegeneration and prevent dementia. For sAD, the search for markers can be realized only in rodent and primate models, that is, in animals that can independently develop the pathology.

Models of early aging animals seem particularly attractive in this case, as they naturally exhibit accelerated aging processes that closely mimic the physiological and molecular changes observed in normal aging, a major risk factor for AD. These models include senescence‐accelerated mouse (SAMP8),^250^ senescence‐accelerated OXYS rats,^251^ and progeroid syndromes in rodents, such as those with defects in DNA repair pathways (e.g., Ercc1‐deficient mice).^252^, ^253^ They often display early‐onset neurodegeneration, neuroinflammation, and metabolic alterations that contribute to AD pathogenesis, making them valuable for studying disease progression and identifying early biomarkers. However, the necessary period and target markers may be missed in the case of rapid alterations in the body's condition. In the case of the genetic form of the disease, it may be more convenient to work on simpler model objects, including zebrafish*, Drosophila*, and *C. elegans*, due to the simplicity of genetic manipulation and analysis.

Furthermore, AD patients often display various comorbidities, including atherosclerosis, diabetes, and hypertension, as well as CNS disorders, such as depression.^254^ All of these comorbid conditions can impact the severity of AD and the progression of the disease, which is not accounted for in animal models, hence creating additional limitations and hindering the detection of pathology by markers.

Another aspect that models might miss by developing pathologies early is age‐related myelin decline, which can exacerbate Aβ pathology and associated behavioral deficits. As myelin ages, it loses its ability to support axons, leading to axonal distress and increased Aβ production through elevated neuronal BACE1 activity and APP C‐terminal fragment (CTF) accumulation. Recent findings^255^ demonstrated that myelin dysfunction in the aging forebrain—modeled by premature white matter aging in myelin mutant mice—leads to microglial activation, impairing Aβ clearance and promoting plaque formation. This genetic model of AD provides a molecular basis for the interaction between the amyloid hypothesis and neuroinflammation in disease progression. These findings further highlight why aging is a major risk factor for AD and suggest a potential link between AD and demyelinating diseases. This also raises an important question: could demyelinating diseases and AD be comorbid, further complicating disease progression and therapeutic strategies?

Thus, novel AD models with comorbidities (AD+ models) are needed to address this problem and facilitate the search for complex treatments. Importantly, some comorbidities themselves can also be considered early markers of AD. For example, clinical depression increases the risk of developing AD 3‐fold,^256^ and the peak of depression progression in AD patients occurs several years before the onset of dementia.^257^ Treatment with antidepressants reduces the development of neurodegeneration and cognitive impairment in AD patients,^258^ collectively indicating a likely common neurobiological basis for these highly prevalent and severe brain diseases. Another example is type 2 diabetes, characterized by insulin resistance and vascular damage, whereas cerebrovascular disease is one of the causes of impaired Aβ clearance, triggering the formation of amyloid plaques in the brain^259^ and thereby directly increasing the risks of AD.^260^ However, the interaction between AD and diabetes is also driven by non‐vascular mechanisms, as insulin modulates synaptic plasticity and long‐term potential through the regulation of glutamatergic N‐methyl‐D‐aspartate receptor (NMDA) receptor expression and α‐amino‐3‐hydroxy‐5‐methyl‐4‐isoxazolepropionic acid (AMPA) receptor internalization.^261^ Indeed, while AD patients demonstrate decreased levels of insulin and IGF‐1 in the brain,^262^ impaired carbohydrate metabolism in the brain is observed at early stages of AD, even before its main symptoms appear,^263^ which can facilitate the early diagnosis of AD.[Table alz70273-tbl-0003]

To model AD together with its comorbidities as “AD+,” a common risk factor can be simulated. For example, clinical data and animal models of chronic stress show overt dysregulation of the corticotropic axis and immune system.^264^, ^265^, ^266^, ^267^, ^268^, ^269^ Chronic stress can also increase the intake of circulating inflammatory cytokines into the brain,^270^ leading to neuroinflammation via the activation of microglia,^256^ and alters synaptic plasticity^271^ and neurotransmission, potentially leading to depression,^272^ itself a risk factor for AD. Neuroinflammation, in turn, contributes directly to AD pathology.^273^ Thus, chronic stress models can reflect the overlapping mechanisms of AD and depression, and reliable stress protocols are adapted to different model species, including rodents,^274^ primates,^275^ zebrafish,^276^ *Drosophila*,^277^ and *C. elegans*,^278^ hence fostering cross‐taxon analyses of these overlapping mechanisms.

Studying the gut–brain axis is also critical for AD modeling. In a mouse AD model, altered gut microbiota has already been reported, while non‐microbial mice demonstrate diminished AD pathology,^279^ suggesting changes in the microbiome as early markers of the disease, clearly meriting further scrutiny. Likewise, culture, ethnicity, and education have recently emerged as additional factors in AD,^280^ calling for addressing them in animal models. While these factors are deeply tied to socioeconomic disparities and societal progress, which are difficult to replicate in animal models, cognitive ability in rodents and primates can still be assessed through various evaluation protocols. For instance, primates, such as chimpanzees, can demonstrate behaviors related to cultural practices, such as ritualized behaviors,^281^ though these models will not fully capture the humanistic aspects of culture and education. However, they may provide insights into how environmental and cognitive factors influence AD progression.

In general, memory impairment may also arise from degeneration of not a vast number of neurons, but rather a part of them, resulting in disorganization of “engrams” (specific ensembles of neurons that encode memories). Yet due to the remaining engrams, there is a possibility to restore memory,^282^ a notion recently corroborated in chickens and mice.^283^, ^284^ Likewise, optogenetic activation of hippocampal memory engram cells recover memory in transgenic *APP/PS1* mice.^285^ Such memory recovery is a two‐step process, first involving engram destabilization (an NMDA‐dependent process) followed by destabilization (accompanied by altered gene expression).^286^ Because memory deficits in AD occur due to destabilization, the engrams become disrupted, and memory is not overwritten. Disrupted NMDA transmission in this case may help avoid retrograde amnesia, explaining therapeutic properties of memantine, an NMDA receptor blocker, in AD.^287^ Thus, the development of animal models of engram alteration in dementia may foster the search for therapies in already developed AD symptoms.

## 4 FUTURE PERSPECTIVES: CHALLENGES IN TRANSLATIONAL AD MODELS AND EMERGING ALTERNATIVES

Despite their limitations, animal models that recapitulate the pathophysiology of AD remain essential for developing preventive, diagnostic, and therapeutic strategies. Transgenic, knockout, and Aβ/tau injection models have provided valuable insights into disease mechanisms, particularly the amyloid hypothesis. Given that Aβ is the major component of AD plaques, it has been a primary target for therapeutic intervention. Many anti‐amyloid antibodies have undergone preclinical studies in mouse models, demonstrating the potential for reducing Aβ aggregation in vivo. Passive immunization with monoclonal antibodies against Aβ, such as mAb158 in Tg‐ArcSwe mice, reduced Aβ protofibrils without affecting insoluble Aβ levels.^288^ Similarly, aducanumab, which selectively binds aggregated Aβ, reduced amyloid burden in aged Tg2576 and APP23 mice.^289^ These studies underscore the utility of Aβ‐depositing mouse models in testing anti‐Aβ therapies. However, despite the promising preclinical results, the translation to human clinical outcomes has been disappointing, as highlighted by the mixed efficacy and controversy surrounding recently approved anti‐amyloid antibodies aducanumab and lecanemab.^290^ Their real‐world effectiveness remains uncertain, emphasizing the need for alternative therapeutic approaches and improved models that better replicate human AD pathology.

To address this translational gap, newer humanized mouse models are being developed. These genetically modified models incorporate human genes, cells, tissues, or organs to enhance relevance to human disease. Humanized mouse models based on sAD risk factors may offer better insights into disease progression and allow for personalized medicine approaches. Furthermore, App knock‐in rat models, such as Apphu/hu rats with a humanized Aβ sequence, are emerging as valuable tools due to their natural App expression and more physiologically relevant pathology.^291^ These rats develop key AD features, including Aβ plaques, microglial activation, synaptic degeneration, and cognitive deficits, closely resembling human disease progression.^292^ While these models provide advancements, challenges in translating findings to the clinic persist, underscoring the need for reliable biomarkers and a deeper understanding of AD heterogeneity.

Beyond rodent models, alternative human‐based systems offer complementary insights into AD pathogenesis. Organoid‐based models, derived from pluripotent stem cells, recapitulate key structural and functional aspects of the human brain. These models have shown potential for drug screening and CRISPR‐Cas9–based genetic editing. However, they still face limitations, including a lack of vascularization, incomplete cell type diversity, and atypical neuronal maturation. Notably, organoids lack essential non‐neural cell types, such as vascular endothelial cells and microglia, which are crucial for AD pathophysiology.^293^ Although organoids do not fully represent the adult human brain, recent studies suggest that they can achieve sufficient neuronal network maturation to model AD‐related dysfunction. Nevertheless, their embryonic‐like state presents a significant challenge for studying late‐onset diseases like AD.

iPSC‐derived models have revolutionized AD research by enabling patient‐specific disease modeling. iPSCs, reprogrammed from somatic cells such as fibroblasts or blood cells, can differentiate into neurons, astrocytes, and oligodendrocytes—cell types central to AD pathology. Most current iPSC studies focus on familial AD (FAD)‐associated mutations due to their well‐defined genetic basis. However, sAD modeling remains challenging because iPSCs do not fully capture age‐related factors that drive late‐onset AD. Advances in iPSC‐based aging models, including transdifferentiation methods that preserve donor epigenetic signatures, offer a promising avenue for better mimicking age‐related neuronal dysfunction.^294^ Additionally, co‐culture and 3D organoid models incorporating multiple brain cell types are enhancing our understanding of AD by highlighting the crucial role of glial–neuronal interactions. Future directions in iPSC research include integrating vasculature and immune components into brain organoids to improve modeling of AD pathogenesis. Similarly, cell therapy, including the administration of mesenchymal stem cells, embryonic stem cells, or induced pluripotent stem cells,^295^, ^296^ holds promise, with several clinical trials currently under way in AD patients. For instance, mesenchymal and iPSCs have been shown to modulate inflammation and promote neuronal growth.^295^

In conclusion, while traditional animal models have contributed significantly to AD research, their translational limitations highlight the need for more physiologically relevant models. Humanized rodent models, zebrafish, organoids, and iPSC‐based systems provide promising alternatives, yet each approach has inherent drawbacks. A multimodal strategy that combines these models, along with advances in bioengineering and genetic editing, may be essential for bridging the gap between preclinical research and clinical application. Thus, as novel genetic, pharmacological, and other experimental models of AD continue to emerge (Table 1), the main challenge in the field is how to best use and co‐apply these models to advance our understanding of AD. While multiple open questions remain (Table 3), it may be necessary to systematically combine and co‐vary multiple models on different model organisms for a correct understanding of mechanisms underlying AD.

**TABLE 3 alz70273-tbl-0003:** Selected open questions related to modeling Alzheimer's disease (AD) across taxa.

Open questions
***Conceptual*** Can memory decline be restored in various AD models? The relationship between stress and AD has been increasingly reported.^297^ How can chronic stress contribute to AD progression in experimental models? What are evolutionarily conserved genetic mechanisms of AD in general, and comorbidity of AD with other mental diseases? How can animal models of AD best dissect these mechanisms? What are evolutionarily conserved *epigenetic* mechanisms of AD in general, and comorbidity of AD with other mental diseases? How can animal models of AD best dissect these mechanisms? What are evolutionarily conserved *epigenomic* (based on RNA modifications) mechanisms of AD in general, and comorbidity of AD with other mental diseases? How can animal models of AD best dissect these mechanisms? Does impaired neurogenesis contribute to the pathogenesis of AD or is it a consequence of the progression of the pathology^298^? Can a high level of neurogenesis aggravate AD pathology? How can animal models of AD best explore these aspects of pathogenesis? How can we model peripheral mechanisms of AD, including systemic inflammation and immune abnormalities^299^? Is it possible to create a model that successfully simulates both core and peripheral symptoms? What newly recognized biomarkers of AD can be additionally used for diagnosis, and for AD modeling in animal models?
***Translational*** Is it possible to use stem cell therapy in animals and humans for AD^300^? In the presence of accompanying diseases, it can be difficult to diagnose AD on the blood markers. How can this problem be overcome and what models can help with that^301^? How does the lifestyle (e.g., physical exercise, diet) influence AD development in humans? How can we model these factors in animals AD studies? How do non‐pharmacological interventions (e.g., diet, physical exercise) contribute to better performance of pharmacological therapy in patients with AD? Can we model this aspect in animals?
***Specific*** What specific shared (across taxa) mechanisms are involved in amyloid beta pathology? What is the role of gut flora in AD? Is the composition of the gut microbiota a gold standard for early identification of AD? How do different diets (e.g., Mediterranean diet vs. Western diet) influence the composition of gut microbiota and AD symptoms? Will correction of lysosomal pathology have an effect on AD pathology? Is it possible to influence mammalian target of rapamycin regulation to reduce the symptoms of AD?
***Applied*** Is it possible to reduce neuroinflammation in AD by affecting the microbiota–gut–brain axis? Sex is important in AD clinical trials.^302^ Can sex‐specific treatments for AD patients be developed? Are estrogen receptors (e.g., estrogen receptor β agonist, as S‐equol) a key pathway for the development of novel AD therapies? Are animals that do not develop AD naturally a suitable model for studying this pathology? The prefrontal cortex is critical in AD pathophysiology.^4^ How can naturally “acortical” animal models (e.g., zebrafish) contribute to our understanding AD pathophysiology beyond “cortial” mechanisms? Can computational models of artificial intelligence and machine learning based on biochemistry, neuroimaging, and life‐tracking data be developed to diagnose and predict the progression of AD^303^?
***Other*** Are anti‐diabetic drugs (e.g., long‐acting analog of glucagon‐like peptide‐1) novel potential therapies for AD? Is it possible to create new non‐canonical classes of therapeutic drugs for AD (e.g., adenosine receptor A_2A_‐targeted, tryptophan metabolite‐based, or vitamin K‐based)^304^, ^305^, ^306^? Okadaic acid is known to cause neuroinflammation and dysfunction of the cholinergic system in rodents.^148^ Does it have the same effect in zebrafish? Do drugs that are used in pharmacological models (e.g., streptozotocin) cause same or opposite effects in different model objects across taxa?

## AUTHOR CONTRIBUTIONS

Study concept: K.B.Y., M.M.K., A.V.K.; design and methodology: K.B.Y., M.M.K., K.V.A., D.S.G.; acquisition of data: M.M.K., K.V.A.; analysis and interpretation of the data: D.S.G., T.G.A., T.S., M.S.A., V.A.Ch., K.B.Y.; drafting of the manuscript: M.M.K., K.V.A., M.S.A.; illustrations: K.V.A.; critical revision of the manuscript for important intellectual content: T.S., L.W.L., L.Y., V.A.Ch., K.B.Y., A.V.K.; supervision: K.B.Y., A.V.K.; procurement of funding: K.B.Y., A.V.K. All authors have read and agreed to the published version of the manuscript. All authors confirmed that they contributed to the intellectual content of this paper and have met the following three requirements: (a) significant contributions to the conception and design, acquisition of data, or analysis and interpretation of data; (b) drafting or revising the article for intellectual content; and (c) final approval of the published article.

## CONFLICT OF INTEREST STATEMENT

The authors declare no conflicts of interest. Author disclosures are available in the supporting information.

## CONSENT STATEMENT

This study did not involve human subjects; therefore, informed consent was not applicable.

## Supporting information



Supporting Information
